# Artificial Intelligence-Enabled End-To-End Detection and Assessment of Alzheimer’s Disease Using Voice

**DOI:** 10.3390/brainsci13010028

**Published:** 2022-12-23

**Authors:** Felix Agbavor, Hualou Liang

**Affiliations:** School of Biomedical Engineering, Science and Health Systems, Drexel University, Philadelphia, PA 19104, USA

**Keywords:** Alzheimer’s disease, dementia, end-to-end, data2vec, large language models, speech and language

## Abstract

There is currently no simple, widely available screening method for Alzheimer’s disease (AD), partly because the diagnosis of AD is complex and typically involves expensive and sometimes invasive tests not commonly available outside highly specialized clinical settings. Here, we developed an artificial intelligence (AI)-powered end-to-end system to detect AD and predict its severity directly from voice recordings. At the core of our system is the pre-trained data2vec model, the first high-performance self-supervised algorithm that works for speech, vision, and text. Our model was internally evaluated on the ADReSSo (Alzheimer’s Dementia Recognition through Spontaneous Speech *only*) dataset containing voice recordings of subjects describing the Cookie Theft picture, and externally validated on a test dataset from DementiaBank. The AI model can detect AD with average area under the curve (AUC) of 0.846 and 0.835 on held-out and external test set, respectively. The model was well-calibrated (Hosmer-Lemeshow goodness-of-fit *p*-value = 0.9616). Moreover, the model can reliably predict the subject’s cognitive testing score solely based on raw voice recordings. Our study demonstrates the feasibility of using the AI-powered end-to-end model for early AD diagnosis and severity prediction directly based on voice, showing its potential for screening Alzheimer’s disease in a community setting.

## 1. Introduction

Alzheimer’s disease (AD), the most common type of dementia, is a growing public health concern which affects over 50 million people globally, expecting to raise to more than 150 million by 2050 [1]. AD is a neurodegenerative disease that involves progressive cognitive declines, including speech and language impairments [2]. A timely diagnosis and stratification are paramount in determining people who are at risk of progressing from healthy to mild cognitive impairment and Alzheimer’s dementia. Early diagnosis and intervention targeting the highly suspected population can effectively reduce the disease burden and save enormous medical resources from those unlikely to progress to dementia [3,4]. 

Thus far, there is no cure available for AD treatment [5]. Current diagnosis for AD mainly relies on paraclinical assessments such as brain imaging or cognitive tests, e.g., Mini-Mental State Examination (MMSE) [6] for evaluating the progression of AD [7,8]. Typically, they are expensive and involve lengthy medical evaluations. Neuroimaging studies [9,10], such as magnetic resonance imaging (MRI), have shown that certain regions of the brain are typically affected in Alzheimer’s disease. These regions include the temporal, parietal, and frontal lobes. In general, the temporal and parietal lobes are more heavily affected in the early stages of the disease, while the frontal lobes become more affected in the later stages. The occipital lobe, which is responsible for processing visual information, is generally not as heavily affected in Alzheimer’s disease. Speech and language function are typically associated with the left temporal lobe, while verbal memory is associated with the hippocampus, which is located in the temporal lobe. However, it is important to note that the brain is a highly complex organ, and these functions can be affected by damage to other areas of the brain as well. In recent years, there has been an increasing interest in developing novel technological approaches to aid in the clinical diagnosis and assessment of AD based on a number of predictors ranging from sociodemographic, cognitive, imaging, and biomedical, to genetic variables. 

Previous work has shown that digital voice contains valuable clinical information for AD diagnosis [11], which thus can be a strong predictor of cognitive impairment in the early stages. Other studies have successfully identified digital voice markers associated with the incidence of dementia among asymptomatic patients which can potentially aid in the early diagnosis of AD [12]. Voice data is easily attainable, and provides an efficient, yet inexpensive means to screen and diagnose AD. Earlier studies on the use of voice data in AD assessment have primarily focused on the acoustic and linguistic information embedded in voice recordings [13,14,15]. Acoustic information such as prosodic, paralinguistic, vocal and lexical features [16] can be extracted from the audio recordings; those acoustic features are typically handcrafted, requiring the task-specific knowledge [12,17,18,19]. Linguistic information, on the other hand, can be extracted from texts either manually transcribed or automatically generated by an automatic speech recognition (ASR) system; those text transcripts can be subsequently used by machine learning or Large Language Models (LLMs) such as Bidirectional Encoder Representations from Transformers (BERT) [20,21] or Generative Pre-trained Transformer 3 (GPT-3) [22]. As a result, these models require textual rather than raw voice input, and are inherently huge, spanning millions to billions of parameters, making them costly to deploy and hence not suitable for frequent testing for continuous monitoring of AD. 

### Aims

**Figure 1:**
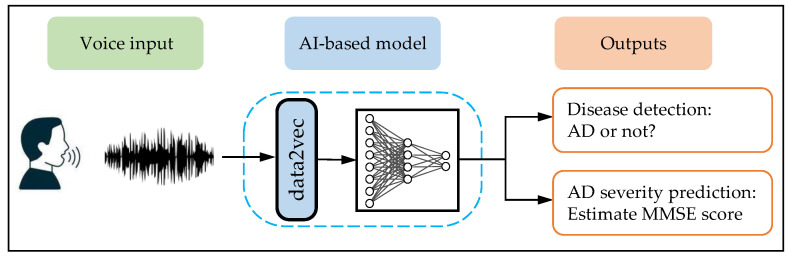
Overview of the AI-powered end-to-end model for AD diagnosis and disease severity prediction from voice. The system takes as input the voice recording from a subject that is converted into the embedding by data2vec, which is then fed into a neural network to determine the AD status and assess the severity of their AD in accordance with MMSE score.

To overcome the above challenges, in this work, we present a new end-to-end AI-powered system (Figure 1) for detection of AD and assessment of AD severity directly through voice. At the core of our proposed approach is the data2vec, a multimodal pre-trained deep learning model that enables the AI system to generate contextualized vector representations (embeddings) directly from raw voice recordings. Such an embedding is a powerful representation of voice that captures not only acoustic information about the audio, but also semantic information embedded in spontaneous voice recordings. The embeddings can then be used by machine learning algorithms such as neural network to infer if a subject has AD and assess the severity of their AD in accordance with MMSE score. 

We tested our AI-based system using the data from the ADReSSo (Alzheimer’s Dementia Recognition through Spontaneous Speech *only*) Challenge [19], a shared task for the systematic comparison of approaches to the detection of cognitive impairment and decline based on spontaneous speech. Using this dataset, we perform two tasks: (1) an AD classification task to discriminate between AD and non-AD subjects and (2) an MMSE regression task to predict the subject’s MMSE cognitive score. Our system was internally evaluated and carefully calibrated on the ADReSSo dataset. It was then validated on an external test dataset from DementiaBank. Our approach aims to deliver a low-cost, unobtrusive, and objective end-to-end AI-based system for AD diagnosis and severity prediction. 

## 2. Materials and Methods

### 2.1. Dataset Descriptions

#### 2.1.1. ADReSSo Dataset

The dataset used in this study is derived from the ADReSSo Challenge [19], which consists of set of speech recordings of picture descriptions produced by cognitively normal subjects and patients with an AD diagnosis, who were asked to describe the Cookie Theft picture from the Boston Diagnostic Aphasia Examination (BDAE) [11,23]. The Cookie Theft picture from the BDAE is a test of visual confrontation naming, which is a measure of a person’s ability to name objects and describe a scene, which can be affected in people with Alzheimer’s disease. The test can help identify difficulty with language and communication, which is often a symptom of AD. However, the BDAE is not a diagnostic tool for Alzheimer’s disease and is only one part of a comprehensive evaluation. Traditionally, screening and tracking the progression of dementia have been carried out using cognitive tests such as the MMSE and the Montreal Cognitive Assessment (MoCA) [24]. These tests are commonly used because they are affordable, easy to administer, and quick to score. Despite its shortcomings in specificity in early stages of dementia, the MMSE is still widely used. 

There are 237 speech recordings in total, with 70/30 split balanced for demographics, resulting in 166 and 71 in the training set and the test set, respectively. In the training set, there are 87 samples from AD subjects and 79 from non-AD (or healthy control) subjects. The datasets were matched so as to avoid potential biases often overlooked in assessment of AD detection methods, including incidences of repetitive speech from the same individual, variations in speech quality, and imbalanced distribution of gender and age. The basic characteristics of the subjects in the training and testing groups are shown in Table 1. In our analysis, a propensity score matching approach [25] was used to match the data. The propensity score was defined based on the probability that an individual with a given age and gender would be treated for AD. To estimate the propensity score, we used a probit regression model to analyze the relationship between the treatment (AD) and the covariates (age and gender). We chose to use a probit model because it resulted in a more balanced dataset compared to using a logistic regression model. This allowed us to better compare the treated and control groups in terms of age and gender. The detailed procedures to match the data demographically according to propensity scores were described in Luz et al. [19]. In the final dataset, all standardized mean differences for the age and gender covariates are <0.001. 

#### 2.1.2. DementiaBank Pitt Database

The DementiaBank Pitt database includes speech recordings from 99 control subjects and 160 AD subjects. Each participant underwent a series of medical and psychiatric examinations to eliminate those cases with confounding conditions that could themselves account for the incidence of dementia [23]. The full database contains multiple speech recordings from different subjects. If a subject has multiple recordings, the subject’s first recording corresponding to their first visit is used as the input to obtain the voice embeddings. The basic characteristics of the subjects are shown in Table 2. 

### 2.2. AI-Based Model

Central to our design is the AI-based model which consists of the pre-trained data2vec, AD classifier, and AD severity predictor (Figure 1). The embeddings generated from data2vec are normalized via a simple z-score before they are fed into the neural network of AD classifier to infer the AD status of the subject and the AD severity predictor to assess their AD severity in accordance with MMSE score, respectively. 

#### 2.2.1. Embeddings from Pre-Trained Model

Data2vec is a general framework for self-supervised learning on multi-modal data [26]. It returns powerful contextualized vector representation, i.e., embedding, directly from either speech, images or textual data. In this work, we use the pre-trained *data2vec-audio-base-960h* base model that was trained in a self-supervised setting for 960 h on the LibriSpeech ASR corpus data [26,27]. The raw audio data is digitalized into floating-point numbers using the Python audio library *librosa* [28] before being processed by the data2vec model. The resultant embedding is taken as the mean of the last hidden state with a vector size of 768. Such embedding after normalization can be directly used as input to a machine learning model for AD detection and for AD severity in accordance with MMSE cognitive score. 

As a benchmark, we also compare our approach with the wav2vec2 [29], a state-of-the-art self-supervised end-to-end ASR system for the AD detection and cognitive score prediction tasks. For the wav2vec2, we use the pre-trained *wav2vec2-base-960h* base model which was also trained on 960 h of LibriSpeech data. For both data2vec and wav2vec2, we access these models using the Huggingface library [30]. 

#### 2.2.2. AD Classifier

The AD classifier consists of a three-layer fully connected feedforward neural network (NN) with a 768-400-1 architecture and the final layer being a sigmoid function. The final output of the classifier is the AD score which is between 0 and 1. A score of 0.5 or more suggests that the subject has AD while a score lower than 0.5 indicates the model classified the input as non-AD. 

#### 2.2.3. AD Severity Predictor

The AD severity prediction is a regression problem, which uses a neural network that is composed of the input layer, two hidden layers, and the output layer in a 768-200-200-1 architecture. The output of the neural network is the estimated MMSE score. A score of 20 to 24 suggests mild dementia, 13 to 20 denotes moderate dementia, and less than 12 indicates severe dementia. As such, the prediction is clipped to a range between 0 and 30. 

### 2.3. Model Development and Validation

#### 2.3.1. Model Training

At each training iteration, a batch of the embeddings is randomly selected as the feature input to the neural network model. For the AD detection task, we minimize the cross-entropy loss with stochastic gradient descent optimizer to train the NN. During training, we use an adaptive learning rate mechanism in which the initial learning rate is kept constant if the training loss keeps decreasing for each iteration. However, if this condition is not met for two consecutive iterations, the learning rate is divided by five and training continues using the new learning rate until the model converges. For the AD severity predictor, we minimize the mean squared error with the Adam optimizer to train the NN. During training, we use a constant learning rate for our AD severity predictor as the Adam optimizer has been shown to have great convergence [31] and hence an adaptive learning rate is not necessary. 

#### 2.3.2. Model Evaluation and Calibration 

We evaluate our model in two ways. The dataset was originally split into 70 percent and 30 percent training and testing set, respectively. We perform internal validation of our AI system using the stratified 10-fold cross-validation (CV) on the entire ADReSSo dataset, that is, both the training and testing dataset are used in the 10-fold CV. To allow for a direct comparison with other published studies, we also train the model on the ADReSSo training set and evaluate the model performance on the unseen, held-out test set. 

Calibration [32,33] has long been studied on uncertainty quantification: a model is calibrated if it assigns meaningful probabilities to its predictions. Concretely, a well-calibrated model should report an 80% confidence when the prediction is correct 80% of the time. Therefore, calibration can give a clear indication whether a method is over- or under-confident in its prediction. Our AD classifier is calibrated using 5-fold cross-validation on the training set. During training, we randomly split the data into 5 subsets and use four for training and the remaining one for calibration. We apply isotonic regression [34] on the raw model output to calibrate the model after training with the four subsets. Specifically, we fit a non-parametric isotonic regressor which minimizes the function $\underset{i}{\sum }{\left(y_{i}-\hat{f_{i}}\right)}^{2}$ where $y_{i}$ is the true label and $\hat{f_{i}}$ is the output of the calibrated classifier for sample *i,* the calibrated probability. Calibration refers to the level of agreement between the observed proportions and predicted probabilities of events. We plot the observed proportions against the predicted probabilities from the calibrated neural network classifier using 5 quantile bins where each bin contains approximately the same number of samples. Specifically, we sort the predicted probabilities, divide them into 5 subgroups equally and find the average of each subgroup. The observed proportions of events were calculated by dividing the number of events by the number of participants. The plot leads to the reliability diagram that provides a visualization of how well-calibrated a model is [32]. A perfectly calibrated model would result in a 45-degree line. The calibrated classifier is then evaluated on the held-out test set and on the external dataset of DementiaBank Pitt corpus to assess the model performance.

### 2.4. Performance Metrics and Statistical Analysis 

The performance metrics we report for the AD detection task include accuracy, precision, recall, and F1 score. Their statistical significance of difference between different machine learning models is assessed by a one-way ANOVA. The root mean squared error (RMSE) and mean absolute error (MAE) scores are used to report for the AD severity prediction. We use the bootstrapping approach [35] to report the 95% confidence intervals for each metric where we randomly perform 5000 resamplings from the set of observations for the evaluation on the test set. We show the receiver operating characteristics (AUC) curves and AUC scores on the test set to measure model discrimination ability. The AUC varies from 0.5 for a non-informative model to 1.0 for a perfect model. While there are several scales for AUC value interpretation dependent on the clinical practice, in general, AUC scores greater than 0.75 are deemed to be clinically useful [36]. The DeLong’s test [37] is adopted to assess the significance of AUCs between our NN and the baseline models described below. 

The calibration is assessed using the Hosmer-Lemeshow goodness of fit test [38] between the predicted probabilities and observed proportions of the events. A *p*-value greater than 0.05 indicates a significant level of agreement between predicted probabilities and observed proportions. We use the Kruskal–Wallis *H*-test to perform a statistical test between the 10-fold CV results of models from the AD severity prediction task to check if the performance improvements are statistically significant. For all the statistical tests reported in this study, a *p*-value less than 0.05 is deemed statistically significant.

### 2.5. Benchmark Studies

We compare the performance of embeddings generated by data2vec to the widely used eGeMAPs acoustic features as well as the wav2vec2. In addition, we also compare our model performance to other machine learning baselines for both the classification and regression tasks, as described below. 

#### 2.5.1. Comparison with Acoustic Features 

Conventional acoustic feature-based approach is used as a benchmark for comparison. The acoustic features considered mainly concern temporal analysis (e.g., pause rate, phonation rate, periodicity of speech, etc.), frequency analysis (e.g., mean, variance, kurtosis of Mel frequency cepstral coefficients) and different aspects of speech production (e.g., prosody, articulation, or vocal quality). In this study, acoustic features are extracted directly from voice using OpenSMILE (open-source Speech and Music Interpretation by Large-space Extraction), a widely used open-source toolkit for audio feature extraction and classification of speech and music signals [39]. We primarily used the extended Geneva Minimalistic Acoustic Parameter Set (eGeMAPS) features due to their potential to detect physiological changes in voice production, as well as theoretical significance and proven usefulness in previous studies [13]. There are, in total, 88 features: the arithmetic mean and coefficient of variation of 18 low-level descriptors (e.g., pitch, jitter, formant 1–3 frequency and relative energy, shimmer, loudness, alpha ratio and Hammarberg index etc), 8 functionals applied to pitch and loudness, 4 statistics over the unvoiced segments, 6 temporal features, and 26 additional cepstral parameters and dynamic parameters. This feature set once obtained can be used directly as inputs to a machine learning model. 

#### 2.5.2. Comparison with Machine Learning Baselines

We compare our model performance with different machine learning models. For AD detection, we use the support vector classifier (SVC), random forest (RF), and logistic regression (LR) as our baselines for the AD diagnosis. SVC and LR both have been used in many healthcare applications while RF has been shown to be very robust on relatively small datasets. 

For the AD severity prediction task, we consider support vector regressor (SVR) and random forest regressor (RFR) as our baseline regression algorithms. SVR has been also widely used in the healthcare domain, whereas the RFR has been utilized in many applications for its robustness due to the ensemble of decision trees. 

## 3. Results

### 3.1. Evaluation of AD Diagnosis

In Table 3, we present the results of 10-fold CV for the AD detection task using data2vec, wav2vec2 embeddings and the acoustic feature set. Our results show that the data2vec embeddings clearly perform better compared to wav2vecv2 and the acoustic feature set. 

**Figure 2:**
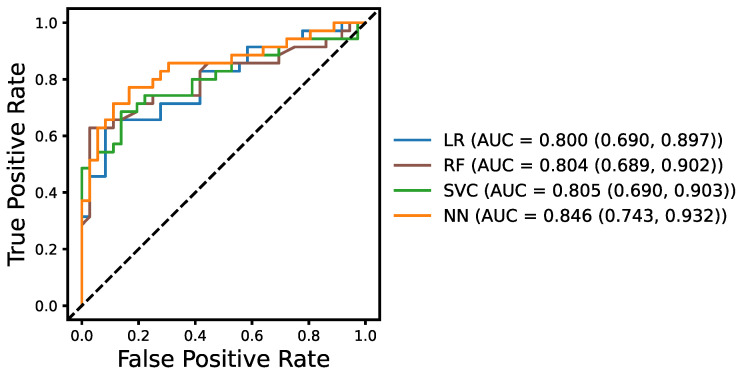
ROC curves for detecting AD using the data2vec from the ADReSSo unseen test set with random forest (RF), support vector classifier (SVC), logistic regression (LR), and neural network (NN) models. The mean AUC and the 95% confidence interval are reported for all four models.

We show in Figure 2 the ROC curves, along with the AUC scores with the 95% confidence interval, for our NN model and the baseline models including LR, RF and SVC. The NN model achieves a relatively high AUC score of 0.846, showing high discriminatory ability. It can also be seen that the NN outperforms all three machine learning baseline models on the ADReSSo unseen test for the AD detection task. The DeLong’s tests for AUC comparison were performed between our NN and each of other prediction models, with the statistical significance of difference only shown between the NN and LR (*p* = 0.0383).

**Figure 3:**
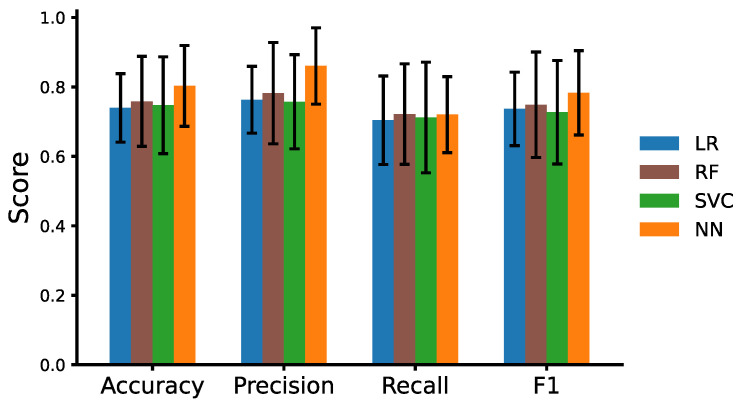
Model Performance for AD detection task using the data2vec embeddings from the ADReSSo unseen test data. The error bar in each metric denotes the 95% confidence interval.

In addition, we evaluate our model performance in terms of accuracy, precision, recall, and F1, and compare it with the results from the three machine learning baseline models for the AD detection task, as shown in Figure 3. The results presented here are from the unseen, held-out test set. We also provide the 95% confidence intervals for each metric reported. The results show that the neural network model again outperforms the other models in terms of accuracy, precision, and F1 scores and has comparable results for the recall. A one-way ANOVA revealed that there was a statistically significant differences between LR, RF, SVC, and NN for accuracy (F = 35.24, *p* = 2.93 × 10^−18^), precision (F = 35.64, *p* = 1.99 × 10^−18^), recall (F = 3.66, *p* = 0.013), and F1 (F = 21.95, *p* = 2.68 × 10^−12^). 

### 3.2. Model Calibration

**Figure 4:**
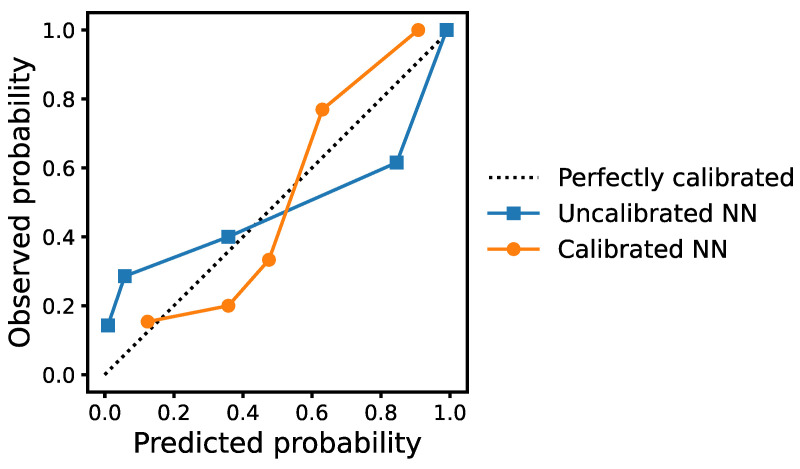
Reliability diagrams of the calibrated and uncalibrated neural network (NN) using isotonic regression.

In Figure 4, we show the calibration plot of the observed proportions and predicted probabilities of the five quantile groups for both the calibrated and uncalibrated NN. It can be seen from the figure that the level of agreement between the observed and predicted groups matches well and a Hosmer-Lemeshow goodness-of-fit test shows that no significant difference exists between the observed proportions and predicted probabilities (*p* > 0.05). 

### 3.3. External Validation

**Figure 5:**
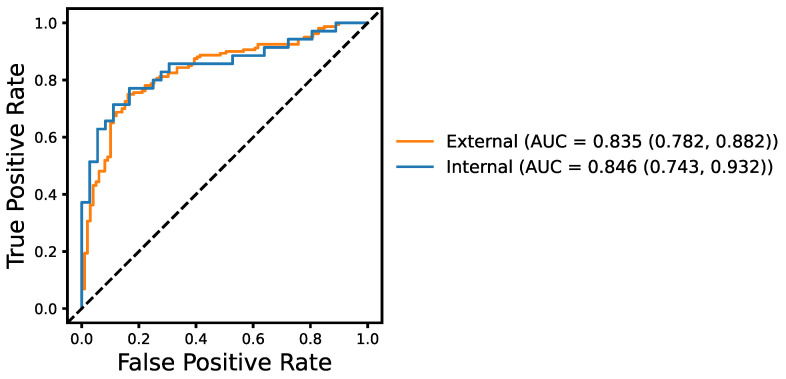
ROC curves and the corresponding AUC scores of the neural network (NN) classifier on the external DementiaBank Pitt dataset and internal unseen ADReSSo test set. 95% confidence interval is also reported for the AUC score.

To assess the generalization ability of our model, we evaluate the model on the external DementiaBank Pitt dataset, never seen during model training and development, and used only for a final test. To allow for direct comparison, we contrast it with the model performance for the internal, held-out test set. Figure 5 shows the ROC curves and AUC scores with the 95% confidence intervals for the model performance on the DementiaBank Pitt dataset and the held-out test set. The model achieves an AUC score of 0.835 on the external dataset as opposed to the AUC score of 0.846 for the internal data, suggesting a good generalizability of our model. 

### 3.4. Evaluation of AD Severity Prediction

Table 4 shows the RMSE and MAE performance of the two regression baseline models and our neural network model. 95% confidence interval for each metric is also reported. We can see that our neural network model outperforms both support vector regressor (SVR) and random forest regressor (RFR) models by having the best root mean square error and mean absolute error scores. Our RMSE score of 4.906 performs favorably over that of the baseline of the competition that is 5.28, as reported by Luz et al. [19] on the ADReSSo Challenge. 

**Figure 6:**
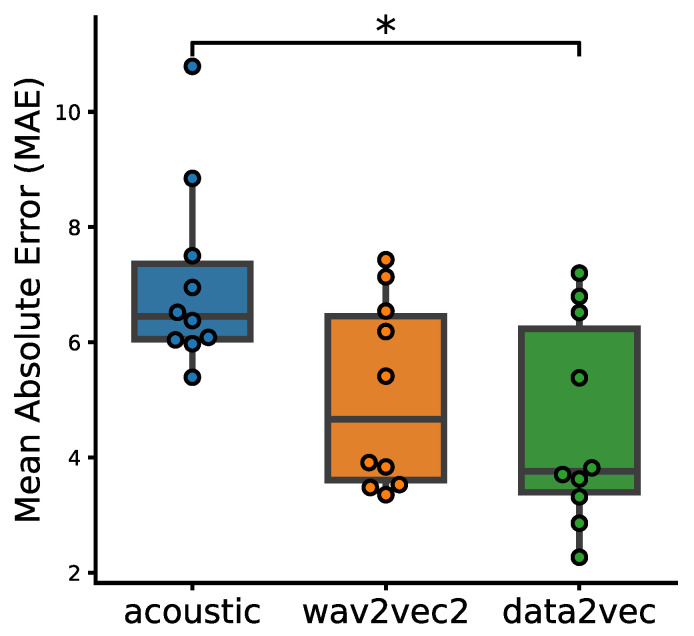
Boxplots of the mean absolute error results for embeddings from data2vec and wav2vec2 and acoustic feature set using the AD severity predictor based on the 10-fold cross-validation. A Kruskal–Wallis *H*-test showed that there is a statistically significant difference between the groups (*H* = 6.9083, *p* = 0.0316). A post hoc Dunn’s test further showed that data2vec is statistically significantly different at *p* = 0.0103 (*).

To compare the performance of the different approaches including data2vec, wav2vec2 and acoustic feature sets for our neural network based AD severity prediction, we show in Figure 6 the boxplots of the three methods based on the 10-fold cross-validation. The plot shows a general decrease from acoustic features to wav2vec2, and further to data2vec embeddings. Data2vec performs the best among the three approaches by having the MAE. A Kruskal–Wallis *H*-test further showed that there is a statistically significant difference between the groups (*H* = 6.9083, *p* = 0.0316). 

## 4. Discussion

The objectives of this work are to develop a new end-to-end AI-powered system for detection of AD and assessment of AD severity directly through voice. The work presented here provides evidence of using the end-to-end AI-powered system to accurately detect AD and predict a subject’s cognitive test score solely based on voice data. The model when trained and tested internally on the unseen test set achieved an AUC of 0.846 on the AD detection task. A further evaluation of the model on an external dataset not used during training showed the model AUC of 0.835, suggesting the good generalizability of our model. Our results also demonstrate that the performance of data2vec is better than wav2vec2 and acoustic features, a finding consistent with previous studies as data2vec predicts contextualized rather than localized representations from the entire input [26]. 

### Strengths and Limitations

Our study has several strengths. First, our approach has the potential to develop and translate a fully deployable AI-powered end-to-end system for early detection of dementia and direct tailored interventions to individual needs. Current diagnosis of AD is primarily based on clinical assessments such as brain imaging, genetics or cognitive tests, which are usually costly in time and money, and bring their own risks (e.g., using radioactive tracers). Language biomarkers have emerged as a medium to detect AD using machine learning techniques, especially LLM [14,18,19,20,22,40,41,42]. However, these models require textual rather than raw voice input, and are inherently large in size, making them expensive to deploy and hence not suitable for frequent testing for continuous monitoring of AD. AD is a disease that progresses slowly, taking years or decades before cognitive decline becomes apparent. It is widely believed that AD is underdiagnosed, particularly in the undeveloped world, but also in the more developed nations. Our AI-powered end-to-end system hence offers a potential solution to develop an easy-to-use, non-invasive, inexpensive voice-based screening tool for the early identification of AD.

Second, our approach has the potential of reducing the cost and duration of AD clinical trials, and hence facilitating drug development. The average cost of treatment and management of AD in the United States was about $305 billion in 2020 with a projected estimate to be over $1 trillion by 2050 [43]. Our AI model could be easily deployed as a web application or even a voice-powered app used in a clinician’s office or even at home for early AD screening. The end-to-end approach is particularly appealing as it allows for continuous monitoring the AD severity in accordance with MMSE scores, which can help clinicians make informed decisions on changes in medication and physical therapy procedures. As such, it can help shorten clinical trials, reduce cost and speed up progress. 

Third, our AI system is well calibrated. Any applications require trustworthy predictions that need to be not only accurate but also able to express their uncertainty. Model calibration and appropriate uncertainty quantification is especially critical for systems to be viable for deployment in high-stakes settings such as the AD diagnosis. Thus, calibration can provide a clear indication whether a method is over- or under-confident in its prediction. Such an approach would serve to add error bars around algorithms, allowing clinicians to find confidence in algorithms. As the AI system becomes further integrated into healthcare environments, the ability to say “I do not know” when uncertain is a necessary capability to enable safe clinical deployment. With this ability, additional human expertise can be sought. 

Several limitations need to be considered when interpreting our findings. First, our study has a relatively small amount of data. For any models to work well, we need to have a large, diverse and robust set of data. Leveraging AI with the growing development of large-scale, multi-modal data such as neuroimaging, speech and language, behavioral biomarkers, and patient information on electronic medical records, will help alleviate the data problem and allow for more accurate, efficient, and early diagnosis [44]. Further research is needed to investigate whether using multi-modal data will improve the performance of the screening method. Second, the bias is involved in model development. It is essential to have voice data in many different languages to protect against this problem and ensure the models work equitably for all patients, regardless of age, gender, ethnicity, nationality and other demographic criteria. Failures in bias consideration could cause disproportionate impact to members of marginalized groups where risks of ethnic conflict arise. Third, the privacy is an important concern in this nascent field, particularly voice data, which can be used to reveal the identity of individuals. Finally, there is a need to establish trust in AI, especially pertinent to the so-called ‘black box’ problem. This often arises in machine learning models where even the developers themselves cannot fully explain, particularly which information are used to make predictions. This can be problematic in clinical practice to explain how a diagnosis of dementia is ascertained and what can determine personalized treatments. Explainable AI aims to address the questions about the decision-making processes. Therefore, it is important to acknowledge that AI is not a replacement for human, but rather provides augmented decision making in driving efficient care and helping make accurate diagnoses. 

## 5. Conclusions

In conclusion, we developed, validated, and tested a novel AI-powered end-to-end model for early AD diagnosis and severity prediction directly based on voice. Our proof-of-concept study provides a simple, accessible, and sensitive community-based screening test that would be useful for early screening and risk assessment before clinical diagnosis. Of note, before the AI-enabled technologies enter mainstream use in aiding the diagnosis of dementia, it is necessary to have rigorous validation from large-scale, well-designed representative studies through multidisciplinary collaboration between AI researchers and clinicians. This will ultimately allow AI to improve early diagnosis, which is crucial to improve quality of life for individuals with dementia. 

## Figures and Tables

**Table 1:** Basic characteristics of the subjects in ADReSSo training and testing (shaded) datasets (M = male. F = female). n/a: not applicable.

Dataset	Age	AD				Non-AD		
		M	F	MMSE (sd)		M	F	MMSE (sd)
Train	[50, 55) [55, 60) [60, 65) [65, 70) [70, 75) [75, 80) [80, 85)	1 4 6 4 5 8 1	0 3 5 17 17 16 0	23.0 (n/a) 17.7 (4.4) 19.2 (6.1) 18.9 (4.2) 17.1 (4.9) 15.8 (5.6) 3.0 (n/a)		1 3 5 8 5 4 1	1 11 8 18 13 1 0	29.0 (1.4) 29.2 (1.0) 29.1 (1.3) 28.9 (1.1) 28.6 (1.3) 29.4 (0.5) 29.0 (n/a)
	Total	29	58	17.4 (5.3)		27	52	29.0 (1.1)
Test	[55, 60) [60, 65) [65, 70) [70, 75) [75, 80)	3 1 3 4 3	3 3 5 4 6	16.8 (4.5) 18.5 (7.6) 19.8 (4.5) 17.6 (6.4) 20.7 (6.7)		3 2 3 5 0	5 5 5 5 3	29.1 (1.4) 28.7 (0.8) 28.8 (0.7) 28.5 (2.5) 29.0 (1.0)
	Total	14	21	18.8 (5.8)		13	23	28.8 (1.5)

**Table 2:** Basic characteristics of subjects for DementiaBank Pitt database. n/a: not applicable.

Age	AD			Non-AD		
	M	F	MMSE (sd)	M	F	MMSE (sd)
[45,50) [50,55) [55,60) [60,65) [65,70) [70,75) [75,80) [80,85) [85,90)	0 2 6 8 8 9 17 4 0	0 0 6 10 22 22 25 12 9	n/a 23.5 (0.5) 18.7 (3.9) 20.4 (4.8) 20.5 (4.5) 18.3 (5.1) 18.6 (4.9) 20.1 (4.2) 20.1 (3.9)	1 4 6 9 9 8 3 1 0	3 4 13 9 15 10 4 0 0	30.0 (0.0) 29.4 (0.7) 29.4 (1.0) 28.9 (1.3) 29.0 (1.0) 28.6 (1.2) 29.1 (0.8) 29.0 (n/a) n/a
Total	54	106	19.4 (4.8)	41	58	29.1 (1.1)

**Table 3:** Classification results for the AD detection task using data2vec embeddings, wav2vec2 embeddings, and eGeMAPs acoustic feature set based on the 10-fold CV with the end-to-end AD classifier. The standard deviation for each metric is reported. Bold indicates the best overall performance for that metric. F1 score is the harmonic mean of the precision and recall.

Embedding	Accuracy	Precision	Recall	F1
eGeMAPs	0.682 (0.101)	0.699 (0.124)	0.704 (0.113)	0.696 (0.097)
wav2vec2	0.721 (0.106)	0.759 (0.151)	**0.711 (0.088)**	0.727 (0.096)
data2vec	**0.730 (0.074)**	**0.778 (0.136)**	0.703 (0.096)	**0.728 (0.071)**

**Table 4:** Root mean squared error (RMSE) and mean absolute error (MAE) results for the performance comparison of data2vec embeddings using the held-out ADReSSo test data. The 95% confidence intervals are reported for both the RMSE and MAE of each model. Bold indicates the overall best score for the metric. SVR: support vector regressor; RFR: random forest regressor; NN: neural network.

Model	RMSE	MAE
SVR RFR NN	4.941 (3.961, 5.887) 6.346 (5.239, 7.410) **4.906 (3.872, 5.912)**	3.784 (3.083, 4.559) 5.059 (4.177, 6.044) **3.493 (2.754, 4.201)**

## Data Availability

All the data are available at https://dementia.talkbank.org (accessed on 14 October 2021).
